# Relative associations of physical activity, social support, and self-control with resilience among vocational college students: a multisite cross-sectional study

**DOI:** 10.3389/fnut.2026.1910091

**Published:** 2026-09-10

**Authors:** Guoguo Zhao, Kai’hong Sun, Jiulong Song, Yuehao Chen, Jian Fu

**Affiliations:** 1 College of Physical Education, Yangzhou University, Yangzhou, Jiangsu, China; 2 Changzhou Vocational Institute of Textile and Garment, Changzhou, Jiangsu, China

**Keywords:** physical activity, predictive modeling, psychosocial resources, resilience, self-control, social support, vocational college students

## Abstract

**Introduction:**

Vocational college students face substantial academic, occupational, and social transition demands, yet the relative importance of modifiable behavioral and psychosocial resources associated with resilience remains unclear.

**Methods:**

This multisite cross-sectional study examined physical activity, social support, self-control, and resilience among 2,251 students recruited by convenience sampling from eight vocational colleges in Jiangsu and Henan Provinces, China. All constructs were assessed using self-report measures. Spearman correlations and covariate-adjusted linear regression were used to compare bivariate and independent associations. Exploratory parallel indirect-association models statistically decomposed the cross-sectional physical activity–resilience association through social support and self-control. Complementary out-of-sample predictive benchmarking compared Ridge regression with gradient boosting, random forest, and support vector regression using repeated nested cross-validation. A personal-resilience outcome was examined as a construct-overlap sensitivity analysis.

**Results:**

Social support showed the strongest adjusted association with resilience (standardized β = 0.587, 95% CI [0.553, 0.621]), followed by self-control (β = 0.344, 95% CI [0.312, 0.376]) and physical activity (β = 0.047, 95% CI [0.018, 0.076]). Positive indirect associations were observed through social support (0.0725, 95% CI [0.0431, 0.1009]) and self-control (0.0653, 95% CI [0.0486, 0.0831]). Ridge regression achieved the numerically highest mean held-out *R*^2^ (0.679), but none of the nonlinear models showed statistically supported improvement beyond the linear benchmark; ordinary least-squares sensitivity analysis produced nearly identical performance (*R*^2^ = 0.678). The broad pattern remained similar when personal resilience was used.

**Discussion:**

In this sample, social support and self-control were substantially stronger independent correlates of resilience than physical activity, while additional nonlinear model complexity offered little out-of-sample predictive gain.

## 1 Introduction

Resilience is a central construct in student mental health because it concerns positive adaptation under adversity rather than simply the absence of psychological symptoms. Developmental accounts characterize resilience as an adaptive process supported by ordinary human systems, while more recent ecological perspectives emphasize that resilient functioning depends on resources distributed across individuals and their social contexts (1–5). This perspective is especially relevant during the transition from education to employment, when academic demands, social expectations, and occupational uncertainty may converge. Across higher education, student mental health is associated with academic, interpersonal, financial, and institutional conditions rather than individual behavior alone (6, 7), while recent meta-analytic evidence shows a substantial inverse association between stress and resilience among college students (8). The vocational college context warrants specific attention because education is closely connected to practical skill development and preparation for the labor market, and evidence from Chinese vocational college students links supportive educational relationships and resilience with mental wellbeing (9, 10). Identifying modifiable resources associated with resilience in this setting is therefore relevant to both student mental health and educational practice.

Three modifiable domains are particularly relevant to this question. Social support can be conceptualized as an external psychosocial resource that provides emotional, informational, and instrumental assistance; the stress-buffering framework proposes that such support can protect functioning under conditions of strain (11, 12). Recent meta-analytic and systematic-review evidence further links perceived social support with mental health, wellbeing, and educational functioning in young people and college students (13, 14), while studies in Chinese vocational and university populations show positive relations between social support and resilience and suggest that coping-related processes may partly account for these associations (15, 16). Self-control represents a complementary internal self-regulatory resource: it involves regulating thoughts, emotions, impulses, and behavior in the service of longer-term goals (17), and has been associated with later health and social outcomes, broad adaptive behavior, and beneficial habits and routines (17–20). Physical activity represents a modifiable behavioral and lifestyle-related resource. Reviews identify psychological, social, behavioral, and neurobiological pathways through which physical activity may support mental health and resilience (21–23), while recent adolescent studies report positive associations with resilience and cross-sectional indirect associations involving self-efficacy, basic psychological needs, and other psychosocial resources (24–27). Taken together, these literatures suggest that resilience may be associated with a configuration of external social resources, internal self-regulation, and health-related behavior rather than with any single domain in isolation.

Despite this converging evidence, two important uncertainties remain. First, physical activity, social support, and self-control are often examined in separate literatures, making it difficult to determine their relative and independent associations when considered simultaneously (14–16, 28). A positive association between physical activity and resilience, for example, may be substantially attenuated once social and self-regulatory resources are taken into account. Second, measurement overlap complicates interpretation of the social-support association. Resilience is commonly conceptualized as a dynamic process supported by interacting personal and contextual resources, including resources within individuals and their broader social environments (29). Because social support measures assess conceptually adjacent resources, a strong association may reflect both substantive covariance and shared content between predictor and outcome. A sensitivity analysis that removes the support-related resilience dimensions is therefore necessary to determine whether the overall pattern persists when this conceptual overlap is reduced (30).

A separate question concerns prediction. Explanatory and predictive modeling address different scientific goals: estimating prespecified associations is not equivalent to demonstrating out-of-sample predictive performance, and stronger prediction does not by itself establish explanatory or causal validity (31, 32). Accordingly, predictive modeling in the present study was not used as a variable-discovery device or as a substitute for conventional association analysis. Instead, it served as complementary out-of-sample benchmarking to test whether additional nonlinear model complexity provided meaningful predictive gain beyond a linear benchmark. Comparing a regularized linear model with gradient boosting, random forest, and support vector regression under repeated nested cross-validation provides a direct test of nonlinear added value while reducing dependence on a single train-test split. Under this framing, failure of a more complex model to improve held-out prediction is itself informative about predictive added value, but it does not prove that the explanatory regression model is correctly specified.

Accordingly, this multisite cross-sectional study had three aims. First, we compared the bivariate, relative, and covariate-adjusted associations of physical activity, social support, and self-control with resilience among 2,251 students recruited from eight vocational colleges in Jiangsu and Henan Provinces. Based on the existing literature, we expected all three domains to be positively associated with resilience, with social support and self-control showing stronger associations than physical activity. Second, we used exploratory parallel indirect-association models to statistically decompose the cross-sectional physical activity-resilience association through social support and self-control; because temporal ordering cannot be established in cross-sectional data, these analyses were treated as noncausal association decompositions rather than tests of mediation mechanisms (33, 34). Third, we conducted complementary out-of-sample predictive benchmarking to determine whether nonlinear models offered additional predictive value beyond a linear benchmark, and repeated the key analyses using a personal-resilience outcome that excluded family support and interpersonal assistance. By separating explanatory, decompositional, predictive, and measurement-sensitivity questions, the study aimed to clarify both the substantive pattern of modifiable correlates and the limits of inference that can be drawn from each analytic component.

## 2 Materials and methods

### 2.1 Study design and participants

**FIGURE 1:**
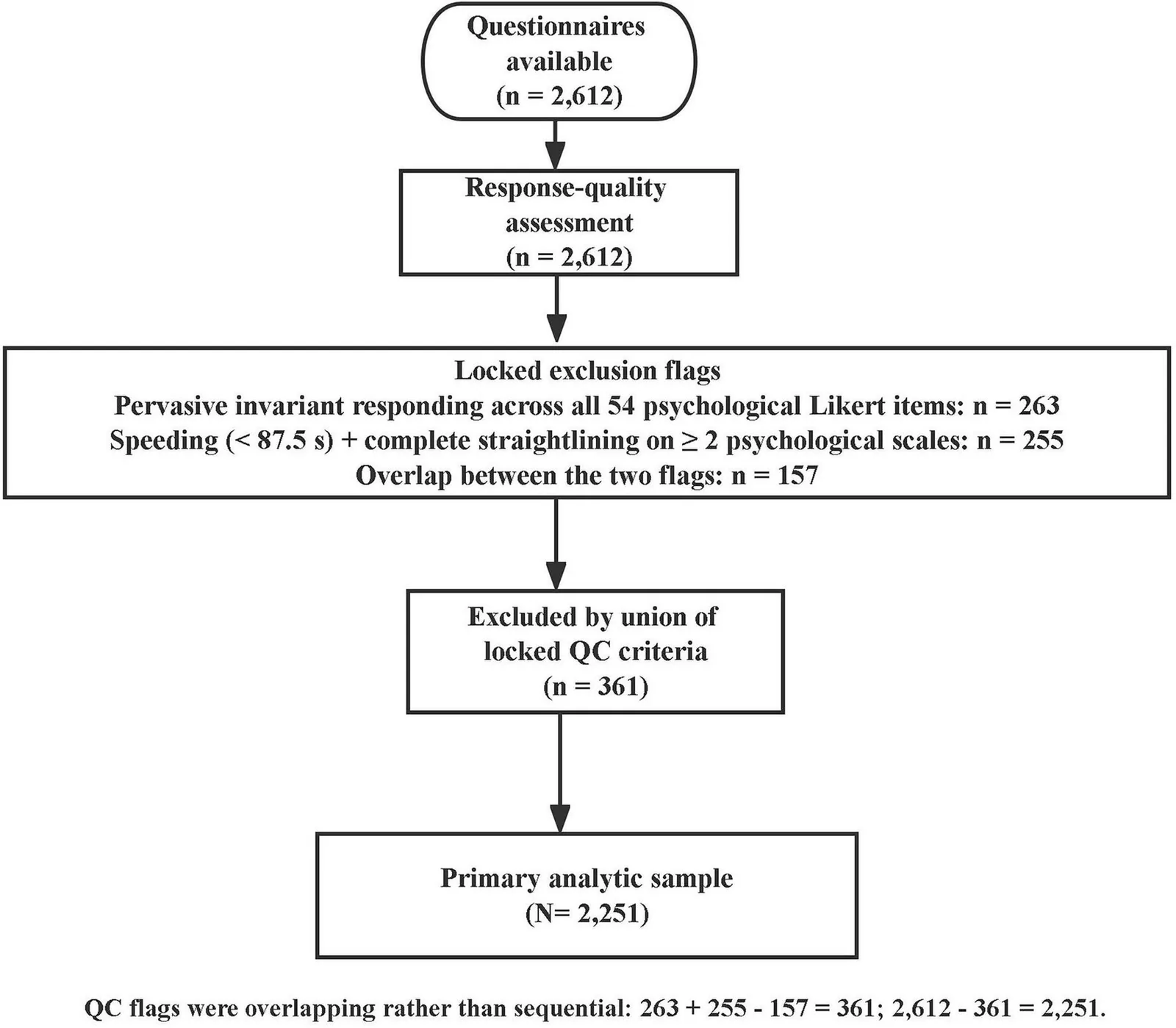
Flow diagram of questionnaire response-quality screening and analytic sample inclusion. The two response-quality exclusion flags were overlapping rather than sequential. Pervasive invariant responding across all 54 psychological Likert items identified 263 records, and rapid completion ( < 87.5 s) combined with complete straightlining on at least two psychological scales identified 255 records; 157 records met both criteria. The union of the two criteria excluded 361 unique records, yielding a final analytic sample of 2,251 participants.

This study used a cross-sectional questionnaire design to examine behavioral and psychosocial correlates of resilience among Chinese vocational college students. The reporting of the study followed the STROBE recommendations for observational research (35). Data were collected between April 17 and May 15, 2025. Participants were recruited by convenience sampling through eight collaborating vocational colleges located in eight cities across two Chinese provinces: Nanjing, Suzhou, Yangzhou, Nantong, and Changzhou in Jiangsu Province, and Zhengzhou, Kaifeng, and Luoyang in Henan Province. Enrolled students participated voluntarily through the collaborating institutions, and written informed consent procedures were conducted in accordance with the approved ethics protocol. A total of 2,612 submitted questionnaires entered response-quality assessment, after which 2,251 participants were retained in the primary analytic sample (Figure 1). The study protocol was approved by the Ethics Committee of the Medical College of Yangzhou University (No. YXYLL-2024-127). Participation was voluntary and anonymous, and participants were informed that they could withdraw from the study at any time. The study was conducted in accordance with the Declaration of Helsinki.

Before substantive analyses were rerun, we locked response-quality criteria based on completion time and item-level response patterns (36). Records were excluded for invariant responding across all 54 psychological Likert items (*n* = 263), or for completion time <0.50 times the sample median (<87.5 s) together with complete straightlining on at least two of the three psychological scales (*n* = 255); 157 records met both criteria. In total, 361 unique records were excluded, leaving 2,251 participants for analysis. Sensitivity analyses using alternative completion-time thresholds are reported in Supplementary Table S1.

### 2.2 Measures

#### 2.2.1 Resilience

Psychological resilience was assessed using the Resilience Scale for Chinese Adolescents, which was developed for Chinese adolescent populations and has been widely used in Chinese youth research (37). The scale contains 27 items across five dimensions: goal focus, emotional regulation, positive cognition, interpersonal assistance, and family support. Items are rated on a five-point Likert scale, and total scores are calculated by summing item responses after reverse-coded items are recoded according to the scoring manual. Higher scores indicate higher psychological resilience. In the present sample, the internal consistency of the total resilience scale was good (Cronbach’s alpha = 0.934).

Because two resilience dimensions, interpersonal assistance and family support, overlap conceptually with social support, a personal-resilience score was constructed for sensitivity analyses. This score retained the individual psychological dimensions of resilience, including goal focus, emotional regulation, and positive cognition, while excluding interpersonal assistance and family support. This sensitivity outcome was used to examine whether the prominence of social support in the primary analyses was attributable to overlap between predictor and outcome measures.

#### 2.2.2 Physical activity

Physical activity was assessed using the Physical Activity Rating Scale-3 (PARS-3), a brief measure commonly used in Chinese student samples to assess exercise intensity, duration, and frequency (38). The physical activity score was calculated using the standard scoring formula: intensity × (duration - 1) × frequency. Higher scores indicate greater participation in physical activity. Because PARS-3 produces a composite exercise score from three components rather than a multi-item latent scale, internal consistency was not used as the primary reliability index for this measure.

#### 2.2.3 Social support

Social support was assessed using the Social Support Scale for University Students, which contains 17 items across three dimensions: subjective support, objective support, and support utilization (39). Items are rated on a five-point Likert scale, with higher scores indicating higher levels of perceived and available social support. The total social support score and the three subdimension scores were used in the analyses. In the present sample, the internal consistency of the total social support scale was excellent (Cronbach’s alpha = 0.976).

#### 2.2.4 Self-control

Self-control was assessed using a simplified self-control scale adapted for Chinese student populations, based on the self-control construct developed in prior psychological research (40). The scale assesses study habits, impulse control, and self-discipline and uses a five-point Likert response format. Items 2, 3, 4, 5, 7, 9, and 10 were reverse-scored, whereas items 1, 6, and 8 retained their original direction, such that higher scores indicated greater self-control. The 10 items were summed to obtain the total self-control score. In the present sample, the internal consistency of the self-control scale was acceptable (Cronbach’s alpha = 0.845).

#### 2.2.5 Demographic variables

Demographic variables included gender, grade, residence, major, and age group. These variables were included as categorical covariates in the adjusted association and indirect-association models and as predictors in the out-of-sample predictive benchmarking. Categorical variables were dummy- or one-hot-coded as appropriate within the corresponding analytic pipeline. Potential common-method effects were additionally assessed using Harman’s single-factor diagnostic based on 57 self-report items or components.

### 2.3 Statistical analysis

Continuous variables were summarized using means, standard deviations, medians, interquartile ranges, and observed ranges. No imputation was required because the variables used in the final analytic dataset were complete for the 2,251 participants retained after response-quality screening. Spearman rank correlations were used to examine the bivariate associations of physical activity, social support, and self-control with resilience. Paired bootstrap resampling with 5,000 iterations was used to obtain 95% confidence intervals for the correlation coefficients (41).

The primary adjusted analysis used multivariable linear regression. Resilience, physical activity, social support, and self-control were standardized, while gender, grade, residence, major, and age group were entered as categorical covariates. HC3 robust standard errors were used to obtain 95% confidence intervals and two-sided *p*-values (42). Independent associations were summarized using standardized regression coefficients, partial R^2^, and incremental R^2^(ΔR^2^). Partial R^2^ for each focal predictor was calculated as the proportional reduction in residual sum of squares when that predictor was added to the corresponding reduced model, whereas ΔR^2^ was calculated as the difference in model R^2^ between the full model and the corresponding model with that predictor omitted. Three prespecified pairwise contrasts between the standardized coefficients of physical activity, social support, and self-control were calculated using the HC3 covariance matrix.

Exploratory parallel indirect-association models were used to decompose the cross-sectional association between physical activity and resilience through social support and self-control. Physical activity was specified as the exposure, social support and self-control as parallel intermediate variables, and resilience as the outcome, with demographic variables included as covariates. Continuous focal variables were standardized before estimation. Specific indirect associations through social support and self-control, the total indirect association, the direct association, and the total association were estimated using 5,000 nonparametric case-bootstrap resamples with percentile-based 95% confidence intervals (43). The analysis was repeated using personal resilience as the outcome for the construct-overlap sensitivity analysis.

Out-of-sample predictive benchmarking was conducted with continuous resilience as the outcome and the same domain-level predictors used in the primary adjusted analysis. Ridge regression served as the linear benchmark and was compared with gradient boosting, random forest, and support vector regression with a radial basis function kernel (44). Models were evaluated using repeated nested cross-validation, with five outer folds repeated five times and five-fold cross-validation for hyperparameter tuning within each outer training set (45, 46). Continuous predictors were standardized and categorical predictors were one-hot encoded within the training folds. The same prespecified predictor set was used across all models without data-driven feature selection. Held-out R^2^ was the primary performance metric, with RMSE and MAE as secondary metrics. Performance was first averaged across the five outer folds within each repeat and then summarized across the five repeats with descriptive 95% confidence intervals. The three nonlinear models were compared with Ridge using paired mean-squared-error differences across the 25 outer evaluations with a corrected repeated-cross-validation test; Holm adjustment was applied across the three nonlinear-versus-Ridge comparisons. Hyperparameter specifications are provided in Supplementary Table S3.

Sensitivity analyses included repeating the predictive benchmark using personal resilience, which excluded the family-support and interpersonal-assistance dimensions, and evaluating an unregularized ordinary least-squares model using the same preprocessing and outer cross-validation splits as Ridge. A supplementary binary-outcome audit retained all prespecified candidate predictors. For this audit, the resilience cutoff was derived from the training-fold median and applied to the corresponding held-out fold, thereby avoiding outcome-threshold information leakage. Results of the binary-outcome audit are reported in Supplementary Table S5.

All analyses were conducted in Python 3.13.5. Data management and numerical operations used pandas 2.2.3 and NumPy 2.3.5; statistical analyses used SciPy 1.17.0 and statsmodels 0.14.6; predictive models and cross-validation were implemented in scikit-learn 1.8.0; and figures were generated using Matplotlib 3.10.8. A fixed random seed of 20260810 was used for all resampling and cross-validation procedures. All statistical tests were two-sided, with *p* < 0.05 considered statistically significant where inferential testing was performed.

## 3 Results

### 3.1 Participant flow and characteristics

Of the 2,612 submitted questionnaires, 361 unique records met the response-quality exclusion criteria, yielding a final analytic sample of 2,251 participants (Figure 1). The sample included 1,528 male students (67.9%) and 723 female students (32.1%). Most participants were first-year students (*n* = 1,417, 62.9%), came from rural areas (*n* = 1,294, 57.5%), and were enrolled in science majors (*n* = 1,745, 77.5%). Participants aged 19–20 years accounted for 70.2% of the sample. Full participant characteristics are presented in Table 1.

**TABLE 1:** Participant characteristics (*N* = 2,251).

Characteristic	Category	*n*	%
Gender	Male	1528	67.9%
	Female	723	32.1%
Grade	First year	1417	62.9%
	Second year	822	36.5%
	Third year	11	0.5%
	Fourth year	1	0.0%
Residence	Urban	957	42.5%
	Rural	1294	57.5%
Major	Humanities	351	15.6%
	Science	1745	77.5%
	Arts	155	6.9%
Age group	< 18 years	13	0.6%
	18 years	420	18.6%
	19 years	865	38.4%
	20 years	715	31.8%
	21 years	197	8.8%
	22 years	36	1.6%
	23 years	3	0.1%
	> 23 years	2	0.1%

### 3.2 Descriptive statistics, reliability, and bivariate associations

Mean scores were 98.90 (SD = 16.07) for resilience, 24.83 (SD = 21.06) for physical activity, 63.25 (SD = 12.75) for social support, and 33.19 (SD = 6.31) for self-control (Table 2). Internal consistency was high for resilience (Cronbach’s α = 0.934), social support (α = 0.976), and self-control (α = 0.845).

**TABLE 2:** Descriptive statistics, reliability, and bivariate associations with resilience (*N* = 2,251).

Variable	Mean (SD)	Median [IQR]	Range	Cronbach’s α	Spearman ρ with resilience	Bootstrap 95% CI	*p*
Resilience	98.90 (16.07)	97 [23]	39–135	0.934	–	–	–
Physical activity	24.83 (21.06)	18 [27]	0–100	–	0.195	[0.154, 0.237]	< 0.001
Social support	63.25 (12.75)	63 [18]	17–85	0.976	0.739	[0.715, 0.761]	< 0.001
Self-control	33.19 (6.31)	32 [7]	14–50	0.845	0.608	[0.576, 0.637]	< 0.001

Values are based on the revised primary analytic sample after the locked response-quality protocol. Spearman correlations are bivariate associations with total resilience; confidence intervals are based on 5,000 bootstrap resamples. All tests are two-sided. PARS-3 physical activity is a composite score and Cronbach’s α is therefore not reported. Higher scores indicate higher levels of each construct.

Resilience was positively associated with social support (Spearman ρ = 0.739, 95% bootstrap CI [0.715, 0.761]), self-control (ρ = 0.608, 95% CI [0.576, 0.637]), and physical activity (ρ = 0.195, 95% CI [0.154, 0.237]); all p < 0.001. Social support showed the strongest bivariate association with resilience, followed by self-control and physical activity. The first unrotated component accounted for 38.40% of the total variance.

### 3.3 Adjusted associations with resilience

**FIGURE 2:**
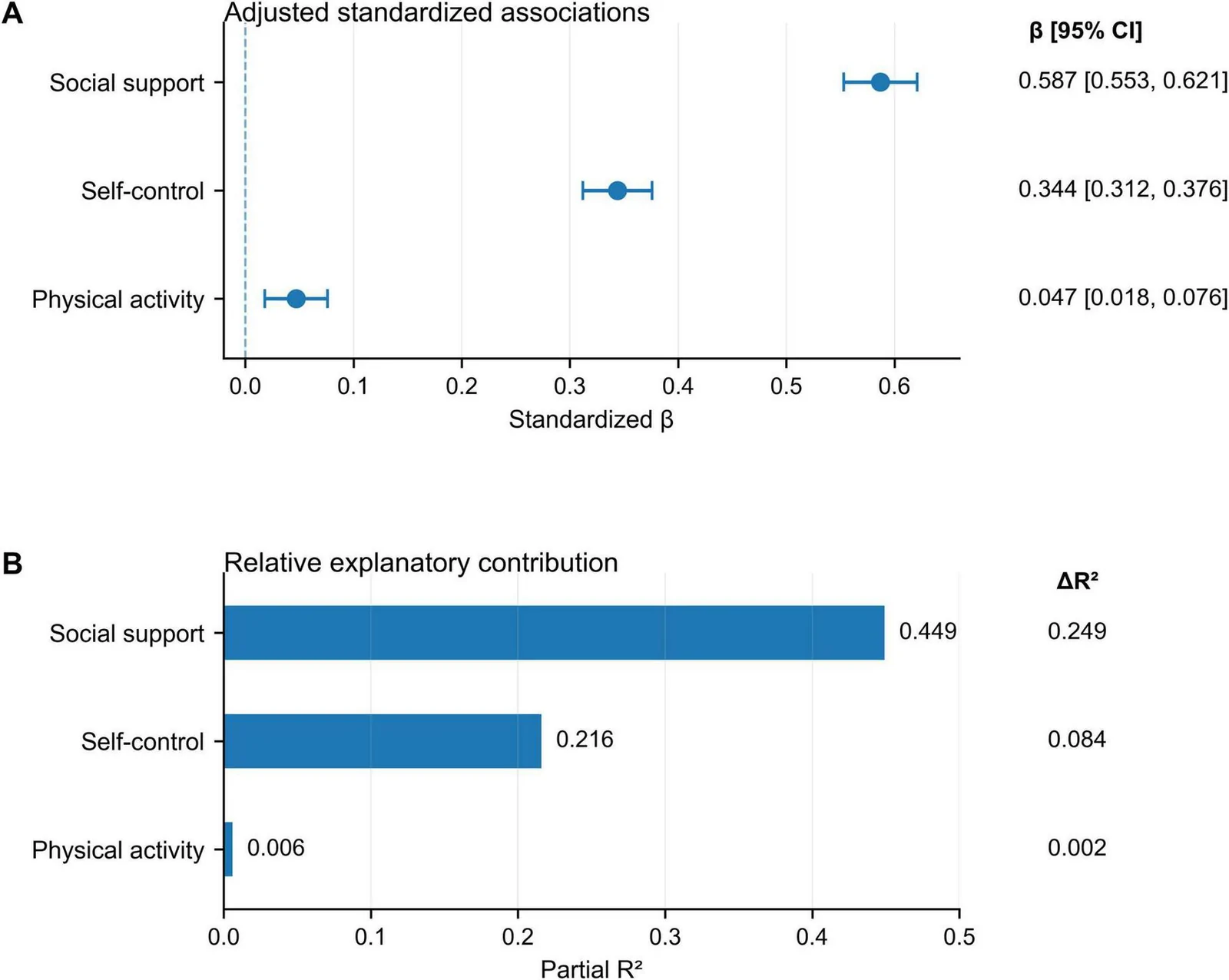
Covariate-adjusted standardized associations of social support, self-control, and physical activity with resilience. **(A)** Standardized regression coefficients with HC3 95% confidence intervals from the primary domain-level model. **(B)** Partial R^2^-values, with ΔR^2^ reported in the adjacent column. The three focal predictors were mutually adjusted, and the model additionally adjusted for gender, grade, residence, major, and age group.

The covariate-adjusted model explained 69.4% of the variance in resilience (*R*^2^ = 0.694; adjusted *R*^2^ = 0.692). Social support showed the largest independent association with resilience (standardized β = 0.587, 95% CI [0.553, 0.621], *p* < 0.001), followed by self-control (β = 0.344, 95% CI [0.312, 0.376], *p* < 0.001). Physical activity remained positively associated with resilience, although the adjusted association was substantially smaller (β = 0.047, 95% CI [0.018, 0.076], *p* = 0.002) (Table 3 and Figure 2).

**TABLE 3:** Adjusted associations of physical activity, social support, and self-control with resilience (*N* = 2,251).

Predictor	Standardized β	HC3 95% CI	*p*	Partial R^2^	Δ R^2^
Social support	0.587	[0.553, 0.621]	< 0.001	0.449	0.249
Self-control	0.344	[0.312, 0.376]	< 0.001	0.216	0.084
Physical activity	0.047	[0.018, 0.076]	0.002	0.006	0.002

Standardized regression coefficients are from the primary domain-level model with total resilience as the continuous outcome. The three focal predictors were mutually adjusted and the model additionally adjusted for gender, grade, residence, major, and age group. HC3 heteroskedasticity-robust confidence intervals are reported. Model *R*^2^ = 0.694; adjusted *R*^2^ = 0.692. Partial *R*^2^ quantifies each predictor’s unique association conditional on the remaining model terms; ΔR^2^ is the decrease in model *R*^2^ when the corresponding focal predictor is removed.

The corresponding partial R^2^ values were 0.449, 0.216, and 0.006, and the corresponding ΔR^2^ values were 0.249, 0.084, and 0.002 for social support, self-control, and physical activity, respectively. Pairwise contrasts of the standardized coefficients showed that the association for social support exceeded that for self-control by 0.243 (95% CI [0.183, 0.302]) and that for physical activity by 0.540 (95% CI [0.495, 0.584]); the association for self-control exceeded that for physical activity by 0.297 (95% CI [0.249, 0.345]); all *p* < 0.001.

The supplementary subdomain model showed a similar overall pattern (Supplementary Table S2). Objective support, subjective support, support utilization, study habits, and impulse control showed positive independent associations with resilience, whereas the confidence interval for self-discipline included zero. Physical activity remained positively but weakly associated with resilience. Variance inflation factors for the substantive predictors ranged from 1.141 to 5.650.

### 3.4 Exploratory indirect associations and construct-overlap sensitivity

In the parallel indirect-association analysis, the standardized indirect association between physical activity and resilience through social support was 0.0725 (95% CI [0.0431, 0.1009]), and the indirect association through self-control was 0.0653 (95% CI [0.0486, 0.0831]). The total indirect association was 0.1378 (95% CI [0.0980, 0.1763]). The direct association was 0.0469 (95% CI [0.0180, 0.0766]), and the total association was 0.1847 (95% CI [0.1389, 0.2316]) (Table 4).

**TABLE 4:** Exploratory cross-sectional indirect associations for total and personal resilience (*N* = 2,251).

Association component	Primary total resilience Estimate (bootstrap 95% CI)	Personal resilience sensitivity Estimate (bootstrap 95% CI)
Indirect association via social support	0.0725 [0.0431, 0.1009]	0.0608 [0.0359, 0.0848]
Indirect association via self-control	0.0653 [0.0486, 0.0831]	0.0698 [0.0520, 0.0889]
Total indirect association	**0.1378 [0.0980, 0.1763]**	**0.1306 [0.0944, 0.1661]**
Direct association	0.0469 [0.0180, 0.0766]	0.0664 [0.0347, 0.0986]
Total association	**0.1847 [0.1389, 0.2316]**	**0.1970 [0.1500, 0.2428]**

Models were adjusted for gender, grade, residence, major, and age group. CIs are percentile bootstrap 95% CIs based on 5,000 resamples. Personal resilience comprises goal focus, emotional regulation, and positive cognition, excluding family support and interpersonal assistance. These cross-sectional decompositions do not establish temporal ordering or causal mediation. Estimates across the two outcome definitions should not be directly compared in absolute magnitude. Bold values indicate the total indirect association and total association estimates for each outcome definition. Bold formatting is used for emphasis only and does not denote statistical significance.

When personal resilience was used as the outcome, excluding the family-support and interpersonal-assistance dimensions, the overall pattern was retained. The indirect associations through social support and self-control were 0.0608 (95% CI [0.0359, 0.0848]) and 0.0698 (95% CI [0.0520, 0.0889]), respectively. The total indirect association was 0.1306 (95% CI [0.0944, 0.1661]), the direct association was 0.0664 (95% CI [0.0347, 0.0986]), and the total association was 0.1970 (95% CI [0.1500, 0.2428]). Thus, the pattern of indirect associations remained similar when support-related dimensions were removed from the resilience outcome. Detailed path coefficients for both outcome definitions are provided in Supplementary Table S7.

### 3.5 Out-of-sample predictive benchmarking

In repeated nested cross-validation, Ridge regression achieved a mean held-out R^2^ of 0.679 (95% descriptive CI [0.677, 0.682]), with an RMSE of 9.070 and an MAE of 6.861. Mean held-out R^2^ values were 0.675 (95% descriptive CI [0.670, 0.680]) for gradient boosting, 0.665(95% descriptive CI [0.663, 0.667]) for random forest, and 0.661 (95% descriptive CI [0.658, 0.663]) for support vector regression (Table 5).

**TABLE 5:** Out-of-sample predictive performance in repeated nested cross-validation.

Model	Mean held-out R^2^ (95% descriptive CI)	RMSE	MAE	Δ R^2^ vs Ridge	Corrected MSE contrast (95% CI)	Holm-adjusted p
Ridge	0.679 [0.677, 0.682]	9.070	6.861	–	–	–
Gradient boosting	0.675 [0.670, 0.680]	9.131	6.951	−0.0043	−1.122 [−4.335, 2.090]	0.478
Random forest	0.665 [0.663, 0.667]	9.264	7.064	−0.0138	−3.563 [−7.788, 0.663]	0.189
SVR (RBF)	0.661 [0.658, 0.663]	9.330	6.964	−0.0186	−4.779 [−9.132, −0.426]	0.098

Performance was estimated using five repeats of five-fold outer cross-validation with five-fold inner tuning. R^2^ confidence intervals are descriptive across the five repeat-level means. Corrected MSE contrasts [MSE(Ridge) - MSE(comparator)] account for repeated-CV dependence; positive values favor the comparator and negative values favor Ridge. Holm adjustment was applied to the three nonlinear-versus-Ridge *p*-values; the corresponding confidence intervals were not multiplicity-adjusted. Negative ΔR^2^ indicates lower mean held-out R^2^ than Ridge.

The corrected MSE contrasts relative to Ridge were −1.122 (95% CI [−4.335, 2.090]) for gradient boosting, −3.563 (95% CI [−7.788, 0.663]) for random forest, and −4.779 (95% CI [−9.132, −0.426]) for support vector regression. The corresponding Holm-adjusted p values were 0.478, 0.189, and 0.098, respectively. After multiplicity correction, none of the nonlinear models showed a statistically supported improvement in out-of-sample prediction over the Ridge benchmark.

### 3.6 Sensitivity analyses of predictive performance

**FIGURE 3:**
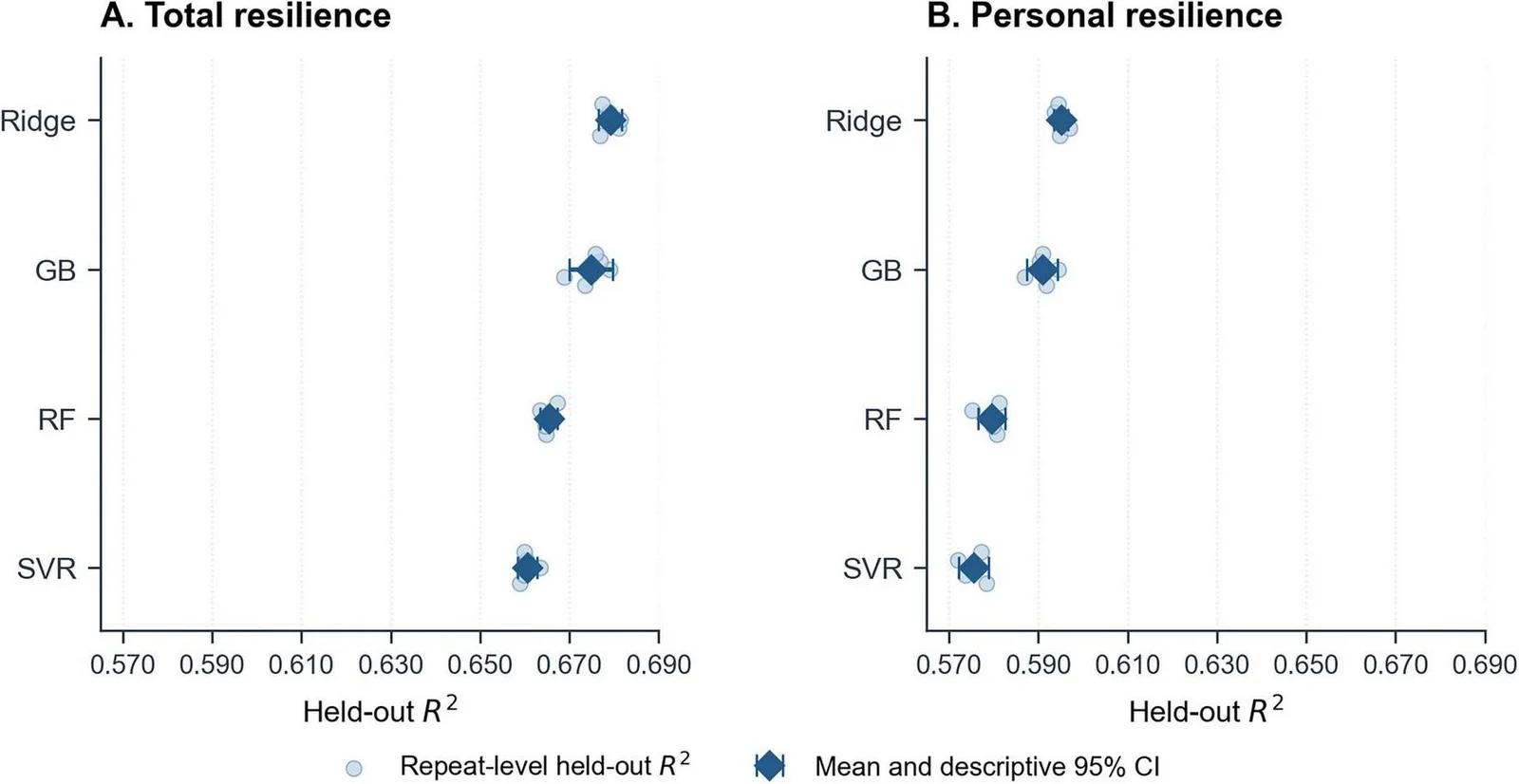
Repeat-level out-of-sample predictive performance for total and personal resilience under repeated nested cross-validation. Small circles show the five repeat-level mean held-out R^2^ values for each model, and diamonds with horizontal error bars show the mean and descriptive 95% confidence interval across repeats. **(A)** Total resilience and **(B)** personal resilience. Ridge, ridge regression; GB, gradient boosting; RF, random forest; SVR, support vector regression with a radial basis function kernel. The figure summarizes internal repeated nested-cross-validation performance and should not be interpreted as external validation.

When personal resilience was used as the outcome, the relative ordering and repeat-to-repeat pattern of model performance remained broadly similar (Figure 3 and Supplementary Table S6). Mean held-out R^2^ was 0.595 (95% descriptive CI [0.593, 0.597]) for Ridge, 0.591 (95% CI [0.588, 0.594]) for gradient boosting, 0.580 (95% CI [0.577, 0.583]) for random forest, and 0.576 (95% CI [0.572, 0.579]) for support vector regression. No nonlinear model showed a statistically supported improvement over Ridge.

The unregularized ordinary least-squares sensitivity analysis produced nearly identical held-out performance to Ridge (mean *R*^2^ = 0.678, 95% descriptive CI [0.676, 0.680]; RMSE = 9.087; MAE = 6.863). The corrected MSE contrast, defined as MSE(Ridge) – MSE(OLS), was −0.309 (95% CI [−0.793, 0.174], *p* = 0.199), indicating no statistically supported difference between the two linear specifications (Supplementary Table S4).

In the supplementary binary-outcome audit retaining all prespecified candidate predictors, random forest and gradient boosting yielded mean AUCs of 0.892 and 0.891, respectively. The corrected random-forest-minus-gradient-boosting AUC difference was 0.0003 (95% CI [−0.0045, 0.0051], *p* = 0.898), providing no evidence that random forest outperformed gradient boosting (Supplementary Table S5).

## 4 Discussion

This study addressed three complementary questions: the relative independent associations of physical activity, social support, and self-control with resilience; the cross-sectional statistical decomposition of the physical activity–resilience association through social support and self-control; and whether additional nonlinear model complexity improved out-of-sample prediction. Social support showed the strongest independent association with resilience, followed by self-control, whereas physical activity remained positively associated but showed a substantially smaller adjusted association and only a small increment in explained variance. The exploratory decomposition indicated that the cross-sectional physical activity–resilience association was statistically shared with both social support and self-control, and the broad association pattern remained when support-related dimensions were removed from the resilience outcome. For prediction, none of the nonlinear models showed statistically supported improvement beyond the linear benchmark. Together, these findings indicate that behavioral, relational, and self-regulatory resources were not equally informative when evaluated within the same framework, while additional nonlinear complexity provided little predictive gain.

The prominence of social support and self-control is consistent with a resource-based view of adaptation. Conservation of resources theory proposes that people seek to acquire, preserve, and mobilize valued resources when confronted with demands, making accessible social resources potentially important for adaptive functioning (47). Recent longitudinal evidence also suggests that social support and resilience are dynamically related over time rather than representing isolated, fixed characteristics (48). Self-control represents a complementary internal resource: contemporary evidence indicates that it can support adaptive behavior through goal prioritization, habit formation, intention–behavior translation, and regulation of competing impulses (49). Importantly, however, the ordering observed here should not be treated as universal. A 2026 cross-sectional study of 1,006 students from nine Chinese universities examined the same broad domains but reported a stronger standardized association of self-control with resilience than of social support, reversing the ordering observed in our sample (50). Differences in educational population, resilience instrument, social-support measure, self-control measure, and model specification provide plausible explanations for this discrepancy. The vocational-college context may also alter the relative salience of interpersonal resources during educational and occupational transition, but this possibility requires direct comparative testing. Measurement must also be considered: the primary resilience scale contains family-support and interpersonal-assistance dimensions, although the persistence of the broad pattern after excluding these dimensions indicates that the prominence of social support was not solely a consequence of shared item content. Because several subdomains were moderately collinear, the secondary subdomain estimates are best interpreted as descriptive refinement rather than as a basis for ranking individual components.

The comparatively small independent association of physical activity deserves a different interpretation from simple dismissal. Its positive bivariate association with resilience is compatible with the broader literature, but its adjusted association became much smaller once social support and self-control were considered simultaneously. One explanation is that physical activity and psychosocial resources are themselves interconnected. A 2024 meta-analysis of college and university students found a positive association between social support and physical activity, with friend support showing a stronger association than family support (51). Longitudinal evidence further indicates that changes in peer support can predict trajectories of physical activity from late adolescence into young adulthood (52). A recent Chinese university study likewise reported associations linking exercise, social support, self-control, and resilience within the same model (50). These findings make directional interpretation particularly difficult: supportive relationships may facilitate participation in physical activity, activity settings may create opportunities for social connection and self-regulation, or both processes may occur together. A second explanation concerns measurement. PARS-3 summarizes exercise intensity, duration, and frequency, but does not distinguish whether activity occurs individually or collectively, competitively or recreationally, or within environments characterized by belonging, challenge, mastery, and interpersonal support. The present findings are therefore compatible with an interconnected behavioral–psychosocial configuration rather than establishing a directional pathway from physical activity through social support or self-control to resilience. The cross-sectional indirect-association estimates should consequently remain association decompositions rather than mechanistic evidence.

The predictive results provide a separate methodological insight. Ridge regression achieved the highest numerical mean held-out performance, but gradient boosting, random forest, and support vector regression did not show statistically supported improvement after accounting for repeated-cross-validation dependence and multiplicity; unregularized OLS performed almost identically to Ridge. The relevant conclusion is therefore not that one linear algorithm was uniquely superior, but that additional nonlinear complexity did not yield reliable predictive gain with the variables available in this study. This finding is consistent with the broader principle that model complexity has scientific value only when it improves generalization to unseen observations. Breiman’s classic distinction between data modeling and algorithmic modeling placed predictive performance on new data at the center of evaluating algorithmic approaches (53). In the present study, the major reproducible predictive signal appears to have been captured adequately by a relatively parsimonious linear representation. This does not imply that the underlying psychological processes are intrinsically linear, nor does it establish that the explanatory regression is fully specified. It indicates only that the tested nonlinear algorithms could not translate their additional flexibility into reliably better held-out prediction for this predictor set and sample.

The findings also suggest a more precise hypothesis for intervention research. Rather than treating increased exercise volume as an isolated route to resilience, physical activity may warrant testing as a context in which relational and self-regulatory resources can also be developed. The observed association between social support and physical activity in university populations supports the plausibility of this proposition (51, 52), although it does not establish that embedding support within exercise will increase resilience. A stronger next-step design would therefore compare physical-activity programs with broadly similar exercise doses but different psychosocial architectures—for example, an activity-only condition versus an otherwise comparable condition that deliberately incorporates peer support, structured goal pursuit, feedback, and self-regulatory practice. The critical question would then shift from whether physical activity is “important” to whether the social and self-regulatory context in which activity occurs changes its association with subsequent adaptation. Such a test would move beyond the accumulation of additional cross-sectional association-decomposition models and directly examine the resource-configuration hypothesis generated by the present findings.

Several strengths increase confidence in the internal coherence of the results, including the multisite sample, explicit response-quality screening, continuous-outcome primary analyses, direct comparison of adjusted associations, repeated nested cross-validation, and sensitivity analyses addressing construct overlap and the choice of linear benchmark. These strengths do not remove important limitations. First, resilience is theoretically defined in relation to adaptation under adversity, yet the present study did not directly establish the nature, severity, or timing of participants’ adversity exposure. The measured outcome is therefore more appropriately interpreted as a self-reported resilience-related capacity or resource profile than as direct evidence of successful adaptation following verified adversity. Second, the cross-sectional design cannot establish temporal ordering among physical activity, social support, self-control, and resilience. Longitudinal studies already suggest that relationships among support, behavior, emotional states, and resilience may unfold dynamically over time (48, 52), reinforcing the need to examine reciprocal rather than exclusively unidirectional processes. Third, all focal variables were self-reported. Shared method variance remains possible, and differences in measurement precision may partly contribute to the smaller coefficient observed for the brief physical-activity index. Fourth, convenience sampling across eight vocational colleges in Jiangsu and Henan increased setting diversity but does not support population-representative estimates for Chinese vocational-college students. Because institution identifiers were not retained in the analytic dataset, institution-level clustering and between-college heterogeneity could not be estimated directly. Finally, repeated nested cross-validation provides rigorous internal validation but not external validation in a geographically independent cohort. Future studies should therefore incorporate explicit measures of adversity, longitudinal or experimental designs, repeated or objective physical-activity measures, institution-level identifiers, and independent external samples.

In conclusion, the present findings refine rather than reject a behavioral account of resilience. Physical activity was positively associated with resilience, but social support and self-control showed substantially stronger independent associations when the three resource domains were considered together. The findings are therefore compatible with a resource-configuration interpretation in which health behavior, relational resources, and self-regulation are considered jointly, while the absence of supported nonlinear predictive gain indicates that additional algorithmic complexity was not necessary to capture the major predictive structure available in these data. The next scientific question is not which single resource should be considered the dominant correlate, but whether longitudinal and experimentally induced changes in these resources—and in the way they are configured—predict subsequent adaptation under demonstrable adversity.

## Data Availability

The de-identified participant-level data are not publicly available because of ethical and privacy considerations. Reasonable requests for access to the data may be directed to Guoguo Zhao, the first author and designated contact for data access, at ggzhao@cztgi.edu.cn. Requests will be considered subject to applicable institutional and ethical requirements. The aggregated results supporting the conclusions of this article are included in the article and Supplementary Material.
